# Irving S. Wright Award: Cellular recycling in aging and disease: The importance of taking out the trash

**DOI:** 10.1093/geroni/igab046.1490

**Published:** 2021-12-17

**Authors:** Malene Hansen

**Affiliations:** Sanford Burnham Prebys Medical Discovery Institute, La Jolla, California, United States

## Abstract

Aging is greatly influenced by quality-control processes that keep the materials inside our cells in proper shape and function. One of these processes is called autophagy, which means "self-eating". This cellular recycling process can digest damaged components to provide new and better parts for the cell. Autophagy plays important roles in many age-related diseases and has been directly linked to aging. In our laboratory, we use the microscopic soil-dwelling round worm C. elegans to understand how autophagy is linked to aging and disease. In this Wright Award seminar, I will discuss our progress on understanding how autophagy is regulated during normal aging and how it may promote a long and healthy lifespan.

